# Taking dietary habits into account: A computational method for modeling food choices that goes beyond price

**DOI:** 10.1371/journal.pone.0178348

**Published:** 2017-05-25

**Authors:** Rahmatollah Beheshti, Jessica C. Jones-Smith, Takeru Igusa

**Affiliations:** 1 Johns Hopkins Global Obesity Prevention Center, Baltimore, Maryland, United States of America; 2 Johns Hopkins Whiting School of Engineering, Baltimore, Maryland, United States of America; 3 Johns Hopkins Bloomberg School of Public Health, Baltimore, Maryland, United States of America; 4 University of Washington School of Public Health, Seattle, Washington, United States of America; University of Southern California, UNITED STATES

## Abstract

Computational models have gained popularity as a predictive tool for assessing proposed policy changes affecting dietary choice. Specifically, they have been used for modeling dietary changes in response to economic interventions, such as price and income changes. Herein, we present a novel addition to this type of model by incorporating habitual behaviors that drive individuals to maintain or conform to prior eating patterns. We examine our method in a simulated case study of food choice behaviors of low-income adults in the US. We use data from several national datasets, including the National Health and Nutrition Examination Survey (NHANES), the US Bureau of Labor Statistics and the USDA, to parameterize our model and develop predictive capabilities in 1) quantifying the influence of prior diet preferences when food budgets are increased and 2) simulating the income elasticities of demand for four food categories. Food budgets can increase because of greater affordability (due to food aid and other nutritional assistance programs), or because of higher income. Our model predictions indicate that low-income adults consume unhealthy diets when they have highly constrained budgets, but that even after budget constraints are relaxed, these unhealthy eating behaviors are maintained. Specifically, diets in this population, before and after changes in food budgets, are characterized by relatively low consumption of fruits and vegetables and high consumption of fat. The model results for income elasticities also show almost no change in consumption of fruit and fat in response to changes in income, which is in agreement with data from the World Bank’s International Comparison Program (ICP). Hence, the proposed method can be used in assessing the influences of habitual dietary patterns on the effectiveness of food policies.

## Introduction

Computational models can serve as powerful tools that complement existing evidence-gathering techniques to develop human health policies [1]. These models can simulate multiple scenarios of policy change at low risk and cost. An important application for such models is in the area of food policy, where we can study the dietary effects of interventions such as food aid or nutritional assistance programs [2–4]. These policy-driven interventions are generally considered as our major tools to improve health outcomes such as obesity and malnutrition [5].

One technique that has been widely used to study food dietary choices is linear programming (LP) [6]. Specifically, LP has been used to generate simulated diets through algorithms that minimize the deviation from average population dietary patterns [7] while satisfying budgetary constraints [8]. LP has been frequently used to assess the economic feasibility of recommended diet guidelines [9, 10] and explore approaches to improve the nutritional impact of food aid policies [11–14].

LP models are appealing, particularly in regard to their mathematical optimization capabilities: they always converge to a unique solution, which is the diet with the minimum deviation from a target diet [15]. Hence, different initial diets that reflect variations in prior eating habits would lead to the same result for the final diet under the LP algorithm. While solution uniqueness is a strength in many respects, in some circumstances it can be a limitation, particularly when history-dependence is of interest. Studies have shown that food preferences develop throughout life in response to food experiences and attitudes [16] starting from neurological and physiological development during childhood [17, 18] or even before the childbirth [19]. In other words, taste preferences and eating habits are formed early in life and persist into adulthood, resulting in dietary habits that are resistant to change. In addition, there has been an increasing realization of the role of the brain reward system in affecting both liking (palatability) and wanting (appetite) of food [20]. These changes affect various hedonic, cognitive and homeostatic aspects of food choice [21]. Thus, habitual dietary choices have important influences that may be highly relevant when modeling responses to policy changes.

In this work, we demonstrate how an agent-based model (ABM) can be used for the analysis of dietary choices under budgetary constraints. An ABM is a software tool wherein a collection of simulated agents, representing individuals, are endowed with characteristics and behaviors that reflect the demographics and behaviors of a population of interest. While ABMs have only recently been introduced in the field of public health nutrition, they are gaining acceptance as a tool that provides insights that complements other existing tools such as LP [22–24]. In the authors’ previous work on agent-based modeling [25], dietary behaviors of individuals from low-income families were examined. The goal of that work was to evaluate the effects of three price metrics (prices in relation to calories, servings and weight) on food choices. It was found that when simulated individuals in the ABM made food decisions based on price per calorie, the results most closely matched the average observed intake of low-income individuals.

In the present paper, we extend this ABM to examine the effects of changes in food budget constraints on dietary choices. We consider two scenarios: in the first, food budgets of low-income individuals are increased to the budget corresponding to the cost of the mean diet for all income groups, as determined by National Health and Nutrition Examination Survey (NHANES). The purpose of this analysis is to examine the extent to which prior dietary habits of low-income individuals would affect dietary behavior when food budget constraints are relaxed. In the second scenario, we analyzed the income elasticity of demand. The income elasticity of demand for each of the main food categories is defined as the percent change in the amount of consumed food in response to a one-percent increase in income. We compute the income elasticities of demand for four food groups, milk, grains, fruit and fats, which are chosen so that they can be compared with the available income elasticities reported by the USDA. Here, the changes in the quantity of the consumed food are considered separately for each food category. Both scenarios examine the effects of food budget changes; the primary difference is that an incremental change is examined in the second scenario.

## Materials and methods

We present our method through a case study based on dietary behaviors of low-income adults in the US. Each agent is assigned an age, gender and BMI, which are randomly generated with means and variances that match the population with the lowest 41% income in the NHANES data set, as described in more detail in the next section. In our model, each simulated individual (agent) adjusts its diet by trying to keep her/his diet as close as possible to the *mean diet*. By mean diet, we refer to the average consumption pattern of our population, given by the average calorie intake for the main food groups (as defined by the USDA). In previous studies of the behavioral influences of food prices, the implicit assumption is that individuals are budget optimizers who consider price-calorie tradeoffs to determine their diets [7–11, 26–28]. This assumption is based on observations that show that people change their *sources* of calories, rather than their total caloric intake, when faced with changes in food budgets or prices [7]. It has also been reported that low-income individuals maintain their identity and self-respect by retaining familiar dietary patterns instead of purchasing the least expensive source of nutrients to achieve a healthy diet [7, 29].

**Fig 1:**
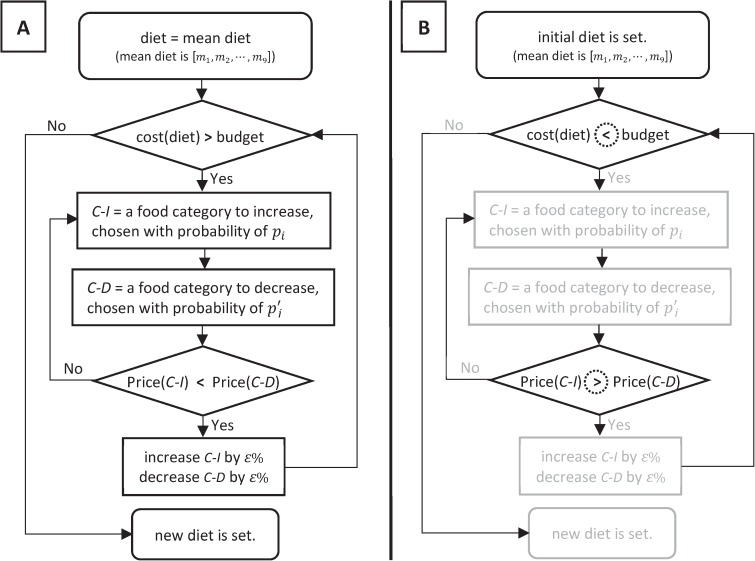
The process used by the agents (individuals as represented in the agent-based model) to update their diet. (A) Normal scenario: agents begin with the mean diet and adjust food intake to meet their food budget constraints. (B) Reversed scenario: agents begin with the diet derived from part (A) and adjust food intake when the food budget is increased to be equal to the cost of the mean diet. The difference between the two scenarios is shown using dotted circles, and boxes similar to the Normal Scenario are shown in gray.

Based on these observations, we follow the process shown in Fig 1A in our ABM to simulate an individual agent’s diet choices under a food budget constraint. Here, an agent’s initial diet is set equal to the mean diet, and is then iteratively and incrementally altered until its cost is less than the agent’s food budget. The iteration begins by randomly choosing an increasing food category (*C-I*) and decreasing food category (*C-D*). The probability *p*_*i*_ of food category *i* being chosen as the increasing food category *C-I* (*i* = 1…*N*, where *N* is the number of food categories) is calculated by:

$$t_{i}=m_{i}*e_{i}$$

$$p_{i}=\frac{t_{i}}{\sum_{k=1}^{N}t_{k}}$$

where *m*_*i*_ refers to the average percentage of calorie intake from food category *i*. The term *e*_*i*_ is the absolute value of the own price elasticity of demand for food category *i*, defined as the percentage change in the demand for food category *i* in response to a one-percent rise in the price of that food category. (The absolute value is used because the own price elasticity of demand is negative.) Variable *t*_*i*_, which is the product of *m*_*i*_ and *e*_*i*_, is divided by its sum to obtain the final set of probabilities *p*_*i*_.

The probability $p_{j}^{\prime }$ of food category *j* being chosen as the decreasing food category *C-D* proceeds with a similar set of expressions:

$$m_{j}^{\prime }=1-m_{j}/\left(N-1\right)$$

$$t_{j}^{\prime }=m_{j}^{\prime }*e_{j}$$

$$p_{j}^{\prime }=\frac{t_{j}^{\prime }}{\sum_{k=1}^{N}t_{k}^{\prime }}$$

It can be seen that $m_{j}^{\prime }$ acts in a complementary manner in relation to *m*_*i*_, in that an increase in *m*_*i*_ results in a decrease in $m_{j}^{\prime }$. It is noted that since $\sum_{i=1}^{n}m_{i}=1$, we will also have $\sum_{j=1}^{n}m_{j}^{\prime }=1$. A higher $m_{j}^{\prime }$ corresponds to a greater likelihood of being chosen as the decreasing category; as before, this likelihood is adjusted by multiplying by the price elasticity *e*_*j*_.

Once the two food categories are chosen, then a candidate for an iterative change in diet is determined by increasing the amount of energy received from food category *C-I* by a small amount (*ε*%), while simultaneously decreasing food category *C-D* by the same amount. This candidate change in diet is accepted only if the price (per calorie) of the first food category is less than the second; otherwise the food category choice algorithm is repeated. In this manner, the total net caloric intake remains unchanged, while the net cost of the diet decreases because of the differential pricing of the two food categories.

This algorithm uses the mean diet as one measure of the preferences while incorporating the influences of the price elasticity of demand, which reflect the willingness of an individual to change food purchases when faced with a price increase. Individuals are more resistant to changing consumption of foods with lower price elasticities. This means that for a food category *i*, a higher *m*_*i*_ (average percentage of calorie) would result in a higher probability of being chosen as the *C-I* category, but this probability is adjusted by multiplying by *e*_*i*_ (absolute value of the own price elasticity of demand) to account for the resistance to change the intake of this food category. It is noted that the use of the mean diet to determine the probability of choosing food categories implicitly considers other factors that affect food choices such as taste, convenience and cultural considerations, since all of these factors have already contributed to the diets that were used to calculate the mean diet.

### Study population, design, and data sources

To assess the performance of our proposed method, we have simulated the food consumption patterns of the adult (>20 years) US population in the year 2001 using our ABM. The size of our agents’ population was set to 201 million, equal to the number of adults in the US on April 2000 [30]. Age and gender of the simulated individuals are assigned based on the US Census data [30] while income and food expenditure data are assigned based on the US Bureau of Labor Statistics (BLS) dataset for 2001 [31]. The non-food portion of food expenditures (such as tips, labor and restaurant taxes) were excluded from the BLS food expenditure values, based on US Department of Agriculture (USDA) datasets on the expenditure of food away from home (FAFH) [32, 33]. This adjustment was necessary because our data on food price (described below) only relates to the price of food itself. In this model, since our focus is on the changes in dietary patterns associated with budgetary factors, we assume that all food is prepared at home.

Each agent is assigned a value for average daily energy intake (EI). Based on the age and gender of each agent, its EI was drawn from the EI distributions as calculated from the NHANES 2001–2002 dataset. These EI values were sampled from 6 normal distributions as reported by Ford and Dietz [34] for the following subpopulations: 2 genders * 3 age groups (i.e., 20–39 yr, 40–59 yr and 60–74 yr). Diets are represented as a list of numbers containing the percentages of EI from the nine major food categories. These nine categories, as indicated by the USDA [35], are: 1) milk and milk products; 2) meat, poultry, and fish; 3) eggs; 4) dry beans, legumes, nuts and seeds; 5) grain products, 6) fruit; 7) vegetables, 8) fats, oils, and salad dressings; and 9) sugars, sweets, and beverages. The mean diet list is denoted as [m_1_, m_2_, …, m_9_] in this paper. To calculate the mean diet, the average proportion of total EI from each of the nine major food categories was derived from the NHANES 2001–02 data [36]. We used the food code variable in the dietary food recall dataset of the same NHANES survey [35] to identify food categories. For adults, the calculated mean diet was: [10.7, 18.6, 1.9, 3.1, 33.4, 4.8, 7.8, 3.0, 16.6]. To avoid generating uncommon and unrealistic diet patterns in our model, the maximum possible value for each category of food is set to the 85^th^ percentile of the NHANES population. Similarly, the minimum value is set to the 15^th^ percentile.

The mean price per calorie ($/100 kcal) of each of the nine food categories is obtained from the work by Drewnowski, et al. [37]. This data was originally calculated using the USDA Food and Nutrient Database for Dietary Studies 1.0 (FNDDS 1.0) [36] and the Center for Nutrition Policy and Promotion (CNPP) food prices database [38], all for the year 2001. Additionally, food price elasticities for all food categories were obtained from Andreyeva et al. [39], except for the beans category, which was obtained from [40].

### Experiments

We begin with a set of validation simulations in which we compare the results of our model with dietary patterns observed in the NHANES datasets. The purpose of this comparison is to ensure that our model is able to correctly simulate the food choices of individuals. We compare the food consumption patterns of our simulated individuals who had the lowest 41% of income against similar individuals in the NHANES 2001–2002 dataset. To identify the individuals with the lowest 41% of income from the NHANES dataset, we used the total family income variable in NHANES. Agents use the process shown in Fig 1A to adjust their diets based on their food budget. It should be noted that the NHANES dataset does not include food expenditure data; hence, we used the BLS dataset [31] for assigning both incomes and food expenditures of our simulated individuals.

The next set of simulations is designed to evaluate the ability of our method to account for the behavioral influence of prior diets. We begin by running our model until the diets of the agents are adjusted according to their food budget (which is essentially the same as our validation simulations described above). We then increase the food budget of the individuals in the lowest 41% income bracket so that the food budgets of these agents are set equal to the cost of mean diet, and then continue running the model until the cost of their diets reaches the new (increased) food budgets. When the food budgets are increased, the diet adjustment follows a similar logic as the process that was presented in Fig 1A. Details of this process are shown in Fig 1B. Instead of trying to decrease the cost of her/his diet, an agent changes her/his diet to increase its overall cost. The values for the increasing (*C-I*) and decreasing (*C-D*) food categories are updated only if the price of *C-I* is greater than *C-D*; otherwise the food category choice algorithm is repeated. After the simulation is complete, we check our results to see if the resulting final diets after the increase in food budget is affected by prior diets. If our model is insensitive to prior diets, then the final diet would be similar to the mean diet; otherwise, the final diet would be significantly different.

We conclude with a set of income elasticity experiments, in which we simulate the income elasticity of demand by using our ABM, and then compare the results to US data. Income elasticity of demand (not to be confused with the price elasticity of demand) refers to the change in the demand of a certain good in response to a 1% increase in income. We use our model to calculate the income elasticity of demand for four food categories: milk, grains, fruit and fats, which were compared with the available income elasticities in the World Bank’s International Comparison Program (ICP), as reported by the USDA [41]. These four categories were selected because these were the only four food categories in our analysis that had corresponding matches in the ICP dataset. We also developed a linear programming (LP) method to compare techniques for simulating the income elasticity of demand. This LP model uses the same set of inputs as our ABM, and is based on the minimization of the distance of the agents’ diets from the mean diet. Other constraints have been also added to this method, including minimum and maximum allowable value for the consumption of each category of food. More details about the LP model are provided in the S1 Text.

The model was developed in the NetLogo environment [42]; the model and its source code is publicly available [43]. Because of the stochastic nature of ABMs, we run our models 100 times, and report the average and confidence intervals of the obtained results. Additional details about our ABM are provided in S2 Text. We formally test the null hypothesis that the simulated diets are identical to the NHANES mean diet, with significance level set to *p* = 0.05. We focused primarily on the individuals with the lowest 41% of income. We report our simulation results for other income percentiles in S3 Text.

## Results

### Comparison with NHANES data

**Fig 2:**
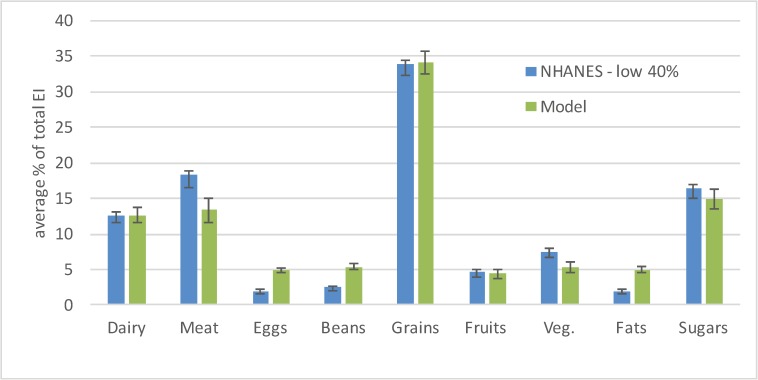
Mean diets of the adult U.S. population with income on or below the 41st percentile, as determined from the NHANES 2001–02 data and simulated by our method. 95% confidence intervals are also shown.

Fig 2 shows the average percentage of energy intake from the nine major categories of foods for the simulated individuals who have the lowest 41% of income. In four of nine cases, the confidence intervals of the simulated results overlap with the average intake of individuals of this income bracket that is recorded in NHANES. We performed a series of t-tests on the simulated results, and at p = 0.05, no significant statistical difference was found between the NHANES data and our simulated results for the Dairy, Grains, Fruits and Sugars food groups.

### Dietary response to increasing budgets

**Fig 3:**
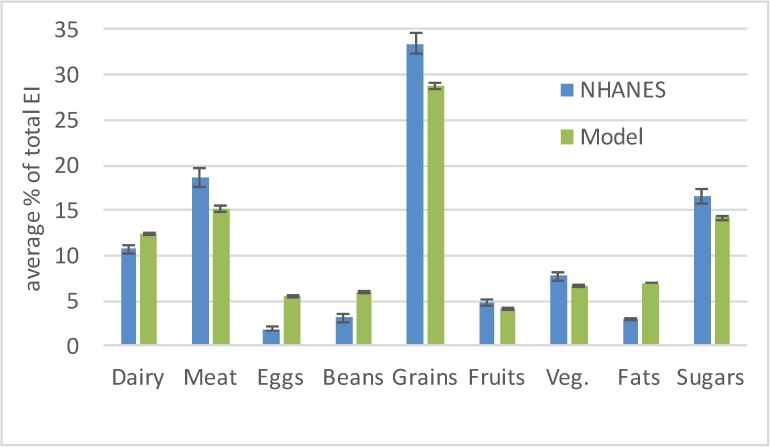
Mean diets of the adult U.S. population as determined from the NHANES 2001–02 data vs. the simulated diets from our model that included the effects of prior eating behaviors. In this scenario, the food budget of the individuals with the lowest 41% of income is increased and is set equal to the cost of mean diet. 95% confidence intervals are also shown.

In this series of simulations, the budgets of agents in the lowest 41% income bracket were increased, and set equal to the cost of the mean diet. This simulates the effects of removing budgetary constraints on low-income individuals. The final diets generated by our method are compared with the NHANES’ mean diet in Fig 3. None of the nine simulated categories had 95% confidence intervals that overlapped with mean diet values, indicating significantly different results between the ABM-generated diet and the mean diet. In fact, after performing t-tests comparing the NHANES data with our model’s results, statistically significant differences were observed in all 9 food groups (p<0.001 for Dairy, Meat, Eggs, Beans, Grains, Fats and Sugars and p<0.05 for Fruits and Vegetables). S4 Text shows the results obtained from a similar experiment using the LP method.

### Income elasticities of demand

**Fig 4:**
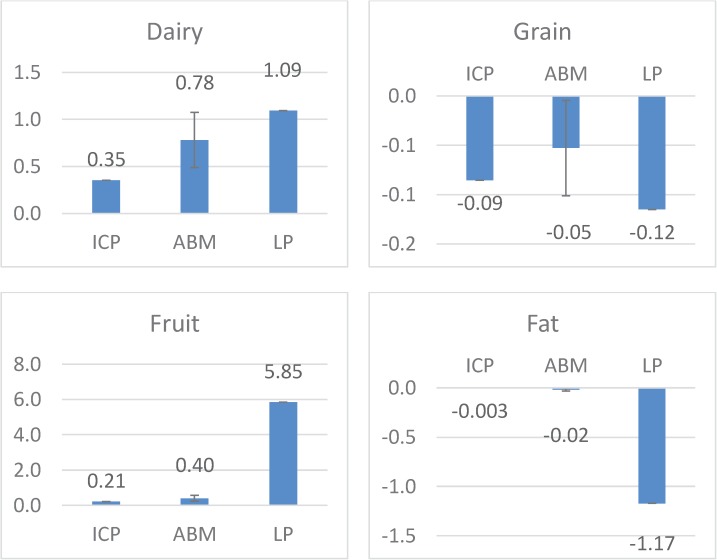
Income elasticity of demand for four different categories of food. The results correspond to the ABM, the LP model and data from the World Bank’s International Comparison Program (ICP).

Fig 4 shows a comparison of the income elasticity of demand, obtained using the ABM, the LP model and data from the World Bank’s International Comparison Program (ICP). The ABM results are close to the actual data with overlapping confidence intervals. More importantly, the ABM results correctly identified the normal goods (i.e., those goods that are consumed at higher levels when the consumer experiences an increase in income, which in this case are milk and fruit), as well as the inferior goods (i.e., those goods that are consumed at lower levels, which in this case are grains and fats). In general, changes in income resulted in exaggerated dietary changes in the LP model, leading to overestimates in the magnitudes of the income elasticities of demand.

## Discussion

Previous work has shown that dietary behaviors are resistant to change because of habits that have been built up over a lifetime [44]. Experiences affecting food choices start from pregnancy, and as children grow, parental modeling and familiarity plays an important role in their developing food preferences [45]. Studies on older adults found that their food choices are strongly influenced by past experiences and beliefs [46]. Life course factors are suggested to be the major component of food choice process, which refer to personal roles and the social, cultural, and physical environments to which a person has previously been exposed [47].

For these reasons, we have developed an ABM for simulating dietary choices that is sensitive to prior dietary habits. We did observe slight increases in the consumption of Fruits and Vegetables after the increase of food budgets, which is in line with other studies on the outcomes of food assistance programs like the Supplemental Nutrition Assistance Program (SNAP) [48]. Nevertheless, our simulated results show that when the income of low-income individuals was increased so that they afford the cost of the mean US diet, their diets did not approach the proportions found in the mean diet. Instead, the diets retained some of the unhealthy characteristics of the former diets under more constrained budgets. In particular, the consumption of Fruits and Vegetables was lower and the proportion of Fats is higher than what is found in the mean diet calculated from NHANES. In a relevant study, it was found that conditional cash transfer programs (a type of welfare assistance program) increased household fruit and vegetable consumption, but also led to excess energy consumption in poor communities in Mexico. An application of our method could be in determining those food categories that require special attention in food policies, as we have demonstrated variable *resistances* of low-income individuals in adopting healthier choices in different food categories. Our method can be also used as a virtual laboratory to test the potential outcomes of a range of candidate interventions, so that we can determine the interventions that maximize desired health outcomes, such as higher consumption of fruits and vegetables.

We have also observed that the income elasticity values of demand that were simulated by our model are closer to the World Bank ICP values as compared with LP results. One important reason for the difference between ABM and LP results is that the LP algorithm always leads to the same diet, regardless of the initial diet used in the procedure. The influence of initial diet in the LP analysis is only on the computation time required to arrive at the final result. It is noted that the uniqueness of the LP result for each level of the income constraint is mathematically appealing, and is an attribute that is suited for many studies of dietary behaviors [3, 8]. For instance, LP models have been proven to be useful because they can isolate the effects of economic and other constraints and examine their impact on food selection [6]. ABMs, which have been used by researchers to study other areas of nutrition [22, 23, 49, 50], can complement LP methods when complex effects of individual and environmental characteristics are of interest. These complex effects can include prior diets, which is the focus of the present study, or other behaviors, such as food dependence on high-fat and high-sugar foods, which have been implicated as a driver of obesity [51]. Hence, policy makers interested in analyzing the potential effects of candidate interventions and policies would benefit from the complementary information they can gain from both LP and ABM models. When compared with other commonly used methods, the only extra data requirement for the proposed method is the price elasticity of demand. These data are easily accessible in reports such as [39] and [40].

The present study has some limitations. The values for the price elasticity of different food categories were determined in terms of consumption patterns of an entire population (including both low- and high-income individuals); however, low-income individuals tend to be more price sensitive, with slightly higher price elasticities. Additionally, our model was designed with a set of simple rules for the dietary choices. While we believe that the current design suffices for the purpose of this work (i.e., assessing the effects of habitual dietary tendencies), in future investigations, more complex models will be needed if additional behaviors are of interest.

## Supporting information

S1 Text Supplemental Text 1. (DOCX)

S2 Text Supplemental Text 2. (DOCX)

S3 Text Supplemental Text 3. (DOCX)

S4 Text Supplemental Text 4. (DOCX)

S5 Text Supplemental NetLogo code. (NLOGO)

## References

[1] Spruijt-Metz D, Hekler E, Saranummi N, Intille S, Korhonen I, Nilsen W, et al Building new computational models to support health behavior change and maintenance: new opportunities in behavioral research. Transl Behav Med. 2015;5(3):335–46. doi: 10.1007/s13142-015-0324-1 26327939 10.1007/s13142-015-0324-1 PMC4537459

[2] Santika O, Fahmida U, Ferguson EL. Development of Food-Based Complementary Feeding Recommendations for 9- to 11-Month-Old Peri-Urban Indonesian Infants Using Linear Programming. J Nutr. 2009;139(1):135–41. doi: 10.3945/jn.108.092270 19056658 10.3945/jn.108.092270

[3] Rambeloson ZJ, Darmon N, Ferguson EL. Linear programming can help identify practical solutions to improve the nutritional quality of food aid. Public Health Nutr. 2008;11. 10.1017/S1368980007000511 17666136

[4] Afshin A, Peñalvo JL, Del Gobbo L, Silva J, Michaelson M, O'Flaherty M, et al The prospective impact of food pricing on improving dietary consumption: A systematic review and meta-analysis. PLoS One. 2017;12(3):e0172277 doi: 10.1371/journal.pone.0172277 28249003 10.1371/journal.pone.0172277 PMC5332034

[5] Hawkes C, Smith TG, Jewell J, Wardle J, Hammond RA, Friel S, et al Smart food policies for obesity prevention. The Lancet. 385(9985):2410–21. 10.1016/S0140-6736(14)61745-1 25703109

[6] Darmon N, Drewnowski A. Contribution of food prices and diet cost to socioeconomic disparities in diet quality and health: a systematic review and analysis. Nutr Rev. 2015;73(10):643–60. doi: 10.1093/nutrit/nuv027 26307238 10.1093/nutrit/nuv027 PMC4586446

[7] Darmon N, Ferguson EL, Briend A. A Cost Constraint Alone Has Adverse Effects on Food Selection and Nutrient Density: An Analysis of Human Diets by Linear Programming. J Nutr. 2002;132(12):3764–71. 12468621 10.1093/jn/132.12.3764

[8] Brimblecombe J, Ferguson M, Liberato SC, O'Dea K, Riley M. Optimisation Modelling to Assess Cost of Dietary Improvement in Remote Aboriginal Australia. PLoS One. 2013;8(12):e83587 doi: 10.1371/journal.pone.0083587 24391790 10.1371/journal.pone.0083587 PMC3877064

[9] Maillot M, Drewnowski A. Energy Allowances for Solid Fats and Added Sugars in Nutritionally Adequate U.S. Diets Estimated at 17–33% by a Linear Programming Model. J Nutr. 2011;141(2):333–40. doi: 10.3945/jn.110.131920 21178090 10.3945/jn.110.131920 PMC3021454

[10] Darmon N, Ferguson E, Briend A. Linear and nonlinear programming to optimize the nutrient density of a population's diet: an example based on diets of preschool children in rural Malawi. Am J Clin Nutr. 2002;75(2):245–53. 11815314 10.1093/ajcn/75.2.245

[11] Dibari F, Diop EHI, Collins S, Seal A. Low-Cost, Ready-to-Use Therapeutic Foods Can Be Designed Using Locally Available Commodities with the Aid of Linear Programming. J Nutr. 2012;142(5):955–61. doi: 10.3945/jn.111.156943 22457396 10.3945/jn.111.156943

[12] Wilson N, Nghiem N, Ryan S, Cleghorn C, Nair N, Blakely T. Designing low-cost “heart healthy bread”: optimization using linear programing and 15-country comparison. BMC Nutrition. 2016;2(1):1–10.

[13] van Dooren C, Tyszler M, Kramer GF, Aiking H. Combining Low Price, Low Climate Impact and High Nutritional Value in One Shopping Basket through Diet Optimization by Linear Programming. Sustainability. 2015;7(9):12837–55.

[14] De Carvalho IST, Granfeldt Y, Dejmek P, Håkansson A. From Diets to Foods: Using Linear Programming to Formulate a Nutritious, Minimum-Cost Porridge Mix for Children Aged 1 to 2 Years. Food Nutr Bull. 2015;36(1):75–85. doi: 10.1177/156482651503600107 25898717 10.1177/156482651503600107

[15] Briend A, Darmon N, Ferguson E, Erhardt JG. Linear Programming: A Mathematical Tool for Analyzing and Optimizing Children's Diets During the Complementary Feeding Period. J Pediatr Gastroenterol Nutr. 2003;36(1):12–22. 12499991 10.1097/00005176-200301000-00006

[16] Mela DJ. Determinants of Food Choice: Relationships with Obesity and Weight Control. Obes Res. 2001;9(S11):249S–55S. 11707550 10.1038/oby.2001.127

[17] Hammond RA, Ornstein JT, Fellows LK, Dubé L, Levitan R, Dagher A. A model of food reward learning with dynamic reward exposure. Front Comput Neurosci. 2011;6:82–. 10.3389/fncom.2012.00082 PMC3468814 23087640

[18] Nader PR, Huang TTK, Gahagan S, Kumanyika S, Hammond RA, Christoffel KK. Next Steps in Obesity Prevention: Altering Early Life Systems To Support Healthy Parents, Infants, and Toddlers. Childhood Obesity. 2012;8(3):195–204. doi: 10.1089/chi.2012.0004 22799545 10.1089/chi.2012.0004

[19] Champagne F, Meaney MJ. Chapter 21 Like mother, like daughter: evidence for non-genomic transmission of parental behavior and stress responsivity. Prog Brain Res. Volume 133: Elsevier; 2001 p. 287–302. 11589138 10.1016/s0079-6123(01)33022-4

[20] Berridge KC. Food reward: Brain substrates of wanting and liking. Neurosci Biobehav Rev. 1996;20(1):1–25. 8622814 10.1016/0149-7634(95)00033-b

[21] Hall KD, Hammond RA, Rahmandad H. Dynamic Interplay Among Homeostatic, Hedonic, and Cognitive Feedback Circuits Regulating Body Weight. Am J Public Health. 2014;104(7):1169–75. doi: 10.2105/AJPH.2014.301931 24832422 10.2105/AJPH.2014.301931 PMC4056226

[22] Zhang D, Giabbanelli PJ, Arah OA, Zimmerman FJ. Impact of Different Policies on Unhealthy Dietary Behaviors in an Urban Adult Population: An Agent-Based Simulation Model. Am J Public Health. 2014;104(7):1217–22. doi: 10.2105/AJPH.2014.301934 24832414 10.2105/AJPH.2014.301934 PMC4056222

[23] Li Y, Zhang D, Pagán JA. Social Norms and the Consumption of Fruits and Vegetables across New York City Neighborhoods. J Urban Health. 2016;93(2):244–55. doi: 10.1007/s11524-016-0028-y 26940705 10.1007/s11524-016-0028-y PMC4835355

[24] Simpson SJ, Raubenheimer D, Charleston MA, Clissold FJ. Modelling nutritional interactions: from individuals to communities. Trends Ecol Evol. 25(1):53–60. doi: 10.1016/j.tree.2009.06.012 19683361 10.1016/j.tree.2009.06.012

[25] Beheshti R, Igusa T, Jones-Smith J. Simulated Models Suggest That Price per Calorie Is the Dominant Price Metric That Low-Income Individuals Use for Food Decision Making. J Nutr. 2016. PubMed Central PMCID: PMC27655757. 10.3945/jn.116.235952 PMC5086791 27655757

[26] Hlaing LM, Fahmida U, Htet MK, Utomo B, Firmansyah A, Ferguson EL. Local food-based complementary feeding recommendations developed by the linear programming approach to improve the intake of problem nutrients among 12–23-month-old Myanmar children. Br J Nutr. 2015:1–11. 10.1017/S000711451500481X 26696232

[27] Masset G, Monsivais P, Maillot M, Darmon N, Drewnowski A. Diet Optimization Methods Can Help Translate Dietary Guidelines into a Cancer Prevention Food Plan. J Nutr. 2009;139(8):1541–8. doi: 10.3945/jn.109.104398 19535422 10.3945/jn.109.104398

[28] Haines PS, Popkin BM, Guilkey DK. Modeling Food Consumption Decisions as a Two-Step Process. Am J Agric Econ. 1988;70(3):543–52.

[29] Dowler E. Poverty, food and nutrition. Why money matters: family income, poverty and children’s lives. London: Save the Children; 2008 p. 34–43.

[30] Age: 2000: Census 2000 brief [Internet]. U.S.Department of Commerce, Economics and Statistics Administration, U.S. Census Bureau. [cited 1 June 2016]. Available from: http://1.usa.gov/1Ur5O2V.

[31] Quintiles of income before taxes, Consumer Expenditure Survey [Internet]. Bureau of Labor Statistics, U.S. Department of Labor. 2001 [cited 1 June 2016]. Available from: http://1.usa.gov/258Whl8.

[32] Dykstra H, Davey A, Fisher JO, Polonsky H, Sherman S, Abel ML, et al Breakfast-Skipping and Selecting Low-Nutritional-Quality Foods for Breakfast Are Common among Low-Income Urban Children, Regardless of Food Security Status. J Nutr. 2016;146(3):630–6. doi: 10.3945/jn.115.225516 26865650 10.3945/jn.115.225516

[33] Relative Prices of Food at Three Stages of the System [Internet]. USDA's Economic Research Service [cited 1 June 2016]. Available from: http://1.usa.gov/1eztJVr.

[34] Ford ES, Dietz WH. Trends in energy intake among adults in the United States: findings from NHANES. Am J Clin Nutr. 2013;97(4):848–53. doi: 10.3945/ajcn.112.052662 23426032 10.3945/ajcn.112.052662 PMC4598942

[35] 2001–2002 National Health and Nutrition Examination Survey (NHANES) [Internet]. Centers for Disease Control Prevention. National Center for Health Statistics. [cited 1 June 2016]. Available from: http://1.usa.gov/23hXuTI.

[36] USDA Food and Nutrient Database for Dietary Studies, v1.0 [Internet]. USDA Agricultural Research Service, Food Surveys Research Group. 2004 [cited 1 June 2016]. Available from: http://www.ars.usda.gov/News/docs.htm?docid=12068.

[37] Drewnowski A. The cost of US foods as related to their nutritive value. Am J Clin Nutr. 2010;92(5):1181–8. doi: 10.3945/ajcn.2010.29300 20720258 10.3945/ajcn.2010.29300 PMC2954450

[38] Development of the CNPP prices database [Internet]. Center for Nutrition, Policy and Promotion. 2008 [cited 1 June 2016]. Available from: http://1.usa.gov/1R9eRQL.

[39] Andreyeva T, Long MW, Brownell KD. The impact of food prices on consumption: a systematic review of research on the price elasticity of demand for food. Am J Public Health. 2010;100(2):216–22. doi: 10.2105/AJPH.2008.151415 20019319 10.2105/AJPH.2008.151415 PMC2804646

[40] Huang KS. Nutrient Elasticities in a Complete Food Demand System. Am J Agric Econ. 1996;78(1):21–9.

[41] Seale JL, Regmi A. Modeling international consumption patterns. Review of Income and Wealth. 2006;52(4):603–24.

[42] Wilensky U. NetLogo. Center for Connected Learning and Computer-Based Modeling, Northwestern University, Evanston, IL; 1999.

[43] The model and its source code will be available on github.com pending approval of this manuscript.

[44] Bellisle F. The determinants of food choice. EUFIC Review. 2005;17(April):1–8.

[45] Savage JS, Fisher JO, Birch LL. Parental Influence on Eating Behavior: Conception to Adolescence. The Journal of Law, Medicine & Ethics. 2007;35(1):22–34. 10.1111/j.1748-720X.2007.00111.x PMC2531152 17341215

[46] Winter Falk L, Bisogni CA, Sobal J. Food Choice Processes of Older Adults: A Qualitative Investigation. J Nutr Educ. 1996;28(5):257–65.

[47] Furst T, Connors M, Bisogni CA, Sobal J, Falk LW. Food Choice: A Conceptual Model of the Process. Appetite. 1996;26(3):247–66. doi: 10.1006/appe.1996.0019 8800481 10.1006/appe.1996.0019

[48] Bartfeld J, Gundersen C, Smeeding T, Ziliak J. SNAP matters: how food stamps affect health and well-being: Stanford University Press; 2015.

[49] Li Y, Berenson J, Gutiérrez A, Pagán JA. Leveraging the Food Environment in Obesity Prevention: the Promise of Systems Science and Agent-Based Modeling. Current Nutrition Reports. 2016;5(4):245–54.

[50] Langellier BA. An agent-based simulation of persistent inequalities in health behavior: Understanding the interdependent roles of segregation, clustering, and social influence. SSM Popul Health. 2016;2:757–69. 10.1016/j.ssmph.2016.10.006 PMC5757936 29349187

[51] Kuo LE, Czarnecka M, Kitlinska JB, Tilan JU, Kvetňanský R, Zukowska Z. Chronic Stress, Combined with a High-Fat/High-Sugar Diet, Shifts Sympathetic Signaling toward Neuropeptide Y and Leads to Obesity and the Metabolic Syndrome. Ann N Y Acad Sci. 2008;1148(1):232–7. 19120115 10.1196/annals.1410.035 PMC2914537

