# Supplementary material for: Taking dietary habits into account: A computational method for modeling food choices that goes beyond price
Source: PLoS One. 2017 May 25;12(5):e0178348. doi: 10.1371/journal.pone.0178348 (PMC5460917; doi:10.1371/journal.pone.0178348)
Supplement: S1 Text — (DOCX) [file pone.0178348.s001.docx]

***LP model***

The goal of an LP-based model is to minimize or maximize an objective function while following a set of constraints represented as linear relations. In our case, the objective function is the difference between each individual’s diet and the mean diet. We follow a similar formulation as the one described by Darmon, et al.[1] We define the objective function, f, as:

| $f=\left\vert(x_{1}-m_{1}) \right\vert/m_{1}+\left\vert(x_{2}-m_{2}) \right\vert/m_{2}+\cdots+\left\vert(x_{9}-m_{9}) \right\vert/m_{9}$ | (1) |
| --- | --- |

where $m_{1}$ to $m_{9}$ are the mean diet elements representing mean caloric intakes for each of the main food categories; $x_{1}$ to $x_{9}$ are the corresponding caloric intakes of the population under study. The absolute distance (|x|) is normalized by dividing by the mean values. The following constraints were defined for the model:

| $\sum_{i=1}^{9} x_{i}=100$ | (2) |
| --- | --- |
| $\sum_{i=1}^{9} {(x}_{i}/100){*EI*Price}_{i}\leq budget$ | (3) |
| $\text{15}^{\text{th}}\text{ percentile} {(x}_{i})< x_{i}<\text{85}^{\text{th}}\text{ percentile} (x_{i})$ | (4) |

Here, the first constraint ensures that the set of $x_{i}$s represent percentages of energy intake, EI, contributed by each food category. The second constraint, $\mathrm{Price}_{i}$, refers to the price (price per calorie) of the i^th^ food category. This constraint is needed to keep the total cost of the diet to be less than the food-budget. As for the proposed ABM, we avoid generation of unrealistic results by considering only $x_{i}$s that are between the 15^th^ and 85^th^ percentiles of food consumption data.

**References:**

1. Darmon N, Ferguson EL, Briend A. A Cost Constraint Alone Has Adverse Effects on Food Selection and Nutrient Density: An Analysis of Human Diets by Linear Programming. J Nutr. 2002;132(12):3764-71.
