# Supplementary material for: Taking dietary habits into account: A computational method for modeling food choices that goes beyond price
Source: PLoS One. 2017 May 25;12(5):e0178348. doi: 10.1371/journal.pone.0178348 (PMC5460917; doi:10.1371/journal.pone.0178348)
Supplement: S2 Text — (DOCX) [file pone.0178348.s002.docx]

**Agent-based model**

In addition to the details that have been presented in the main text for the process of designing our agent-based model (ABM), some other details are provided here. The source-code of our ABM is also available as another appendix. Table 1 lists some of the important implementation details of our ABM.

Table 1. Implementation details for our ABM

| Demographic info source | US Census data for 2001 [1] |
| --- | --- |
| Food budget source | Bureau of Labor Statistics (BLS) [2] |
| Food price source | Drewnowski et al. [3] |
| Food categorization source | US Department of Agriculture (USDA) [4] |
| Mean diet source | NHANES 2001-02 [5] |
| Number of agents | 201 million |
| Period of simulation | 30 days |
| Environment for developing the ABM | Netlogo [6] |

Every ABM consists of a set of agents, along with a set of rules that determine the actions of these agents. In our model, agents are basically simulated individuals who decide on their diets by following the process shown in Figure 1A in the main text. In this process, an agent’s initial diet is set to the mean diet, and is then iteratively changed until its cost is less than or equal to the agent’s food budget. The iterative changes in the diet is performed by increasing the amount of energy received from a certain food category (shown by C-I in that figure) by a small amount ($\varepsilon$), and decreasing another food category by the same amount. While the total net calorie intake stays the same, the net cost of the diet decreases because of the differential pricing of the two food categories. The probability of an increase in a food category is determined by the percentage of each food category in the mean diet, and also the price elasticity of demand for that category. The equations for assigning these probabilities are presented in the main paper.

A detailed description of the data preparation process is provided in our another work [7]. We have performed each simulation over a one-month (30 days) period, during which we assume that there are no changes in food prices and agent body weights. On every day of simulation, each agent starts by an initial diet equal to the mean diet, and then modifies that diet until it has a lower cost than the agent’s food budget. Depending on the experiment, an agent uses either the process described in Fig. 1A or 1B in the main text to update its diet. The results that are reported are based on the average food consumption over this time period. Models are run 100 times (100 simulations, each of 30-day duration). The agents’ population size was set to 201 million, equal to the number of adults (age > 20 yr) in the US, on April 2000 [1]. Our agent-based model was implemented in the NetLogo environment [6].

**References**

1. Age: 2000: Census 2000 brief [Internet]. U.S.Department of Commerce, Economics and Statistics Administration, U.S. Census Bureau. [cited 1 June 2016]. Available from: <http://1.usa.gov/1Ur5O2V>.

2. Quintiles of income before taxes, Consumer Expenditure Survey [Internet]. Bureau of Labor Statistics, U.S. Department of Labor. 2001 [cited 1 June 2016]. Available from: <http://1.usa.gov/258Whl8>.

3. Drewnowski A. The cost of US foods as related to their nutritive value. Am J Clin Nutr. 2010;92(5):1181-8. doi: 10.3945/ajcn.2010.29300.

4. USDA Food and Nutrient Database for Dietary Studies, v1.0 [Internet]. USDA Agricultural Research Service, Food Surveys Research Group. 2004 [cited 1 June 2016]. Available from: <http://www.ars.usda.gov/News/docs.htm?docid=12068>.

5. 2001-2002 National Health and Nutrition Examination Survey (NHANES) [Internet]. Centers for Disease Control Prevention. National Center for Health Statistics. [cited 1 June 2016]. Available from: <http://1.usa.gov/23hXuTI>.

6. Wilensky U. NetLogo. Center for Connected Learning and Computer-Based Modeling, Northwestern University, Evanston, IL; 1999.

7. Beheshti R, Igusa T, Jones-Smith J. Simulated Models Suggest That Price per Calorie Is the Dominant Price Metric That Low-Income Individuals Use for Food Decision Making. J Nutr. 2016. doi: 10.3945/jn.116.235952. PubMed Central PMCID: PMC27655757.
