# Supplementary material for: Taking dietary habits into account: A computational method for modeling food choices that goes beyond price
Source: PLoS One. 2017 May 25;12(5):e0178348. doi: 10.1371/journal.pone.0178348 (PMC5460917; doi:10.1371/journal.pone.0178348)
Supplement: S3 Text — (DOCX) [file pone.0178348.s003.docx]

***Additional Results***

The results that have been presented in the main text were related to the population with the lowest 40% of income (based on the NHANES dataset). In the following, the results from a similar set of experiments on the population with the lowest 23% and 13% of income are reported.

Fig 1. **Mean diets of the adult U.S. population with income on or below the 23^rd^ percentile, as determined from the NHANES 2001-02 data and simulated by our method.** 95% confidence intervals are also shown.

Fig 2. **Mean diets of the adult U.S. population with income on or below the 13^th^ percentile, as determined from the NHANES 2001-02 data and simulated by our method.** 95% confidence intervals are also shown.

Fig 3. **Mean diets of the adult U.S. population as determined from the NHANES 2001-02 data vs. the simulated diets from our model that included the effects of prior eating behaviors.** In this scenario, the food budget of the individuals with the lowest 23% of income is increased and is set equal to the cost of mean diet. 95% confidence intervals are also shown.

Fig 4. **Mean diets of the adult U.S. population as determined from the NHANES 2001-02 data vs. the simulated diets from our model that included the effects of prior eating behaviors.** In this scenario, the food budget of the individuals with the lowest 13% of income is increased and is set equal to the cost of mean diet. 95% confidence intervals are also shown.

|  |  |
| --- | --- |
|  |  |

Fig 5.  **Income elasticity of demand for four different categories of food**. The results correspond to the ABM, the LP model (for the lowest 23% of income) and data from the World Bank’s International Comparison Program (ICP).

|  |  |
| --- | --- |
|  |  |

Fig 6. **Income elasticity of demand for four different categories of food**. The results correspond to the ABM, the LP model (for the lowest 13% of income) and data from the World Bank’s International Comparison Program (ICP).
