# Supplementary material for: Taking dietary habits into account: A computational method for modeling food choices that goes beyond price
Source: PLoS One. 2017 May 25;12(5):e0178348. doi: 10.1371/journal.pone.0178348 (PMC5460917; doi:10.1371/journal.pone.0178348)
Supplement: S4 Text — (DOCX) [file pone.0178348.s004.docx]

**Comparisons between our agent-based model and the linear programming model**

Figure 2 shows the average percentage of energy intake for the nine major categories of foods for the simulated individuals with food budgets equal to the 41^st^ or lower percentiles of food expenditures. The sum of the nine absolute differences between the NHANES data and the simulated results was 17.85 for the ABM and 24.55 for the LP model. The implementation details for the ABM and LP models are presented in other appendices.

Fig 1. Mean diets of the adult U.S. population with food budgets on or below the 41^st^ percentile of food expenditures, as determined from the NHANES 2001-02 data and simulated by the ABM and LP models. 95% confidence intervals are shown for the NHANES and ABM results; LP gives a unique value for each food group.

As described in the main text, in a second experiment we have studied how much our ABM results will be different from the mean diet when our simulated agents can afford the cost of mean diet. We have observed that ABM results ae siginificantly different from the actual mean diet as caculated by the NHANES. For the case of LP model results, we already know that the LP algorithm will always minimize the difference between the generated diet and the mean diet given the budgetary constraint. It is clear (and is easily verified computationally) that the final diet generated by the LP algorithm after increasing income from to lowest 41% of the income distribution would yield the NHANES mean diet. In other words, the null hypothesis that the final diet generated by the LP is identical to the NHANES mean diet is never rejected. The LP model will always generate a final diet that is equal to the mean diet, regardless of the initial conditions for the individual diet.
